# Post-Translational Modification of HMGB1 Disulfide Bonds in Stimulating and Inhibiting Inflammation

**DOI:** 10.3390/cells10123323

**Published:** 2021-11-26

**Authors:** Ulf Andersson, Kevin J. Tracey, Huan Yang

**Affiliations:** 1 Department of Women’s and Children’s Health, Karolinska Institute, Karolinska University Hospital, 17176 Stockholm, Sweden; 2 Institute for Bioelectronic Medicine, The Feinstein Institutes for Medical Research, 350 Community Drive, Manhasset, NY 11030, USA; kjtracey@northwell.edu (K.J.T.); hyang@northwell.edu (H.Y.)

**Keywords:** HMGB1, RAGE, TLR4, DAMP, SIRT1, α7-nicotinic acetylcholine receptor, nociceptor, inflammation, cancer, COVID-19

## Abstract

High mobility group box 1 protein (HMGB1), a highly conserved nuclear DNA-binding protein, is a “damage-associated molecular pattern” molecule (DAMP) implicated in both stimulating and inhibiting innate immunity. As reviewed here, HMGB1 is an oxidation-reduction sensitive DAMP bearing three cysteines, and the post-translational modification of these residues establishes its proinflammatory and anti-inflammatory activities by binding to different extracellular cell surface receptors. The redox-sensitive signaling mechanisms of HMGB1 also occupy an important niche in innate immunity because HMGB1 may carry other DAMPs and pathogen-associated molecular pattern molecules (PAMPs). HMGB1 with DAMP/PAMP cofactors bind to the receptor for advanced glycation end products (RAGE) which internalizes the HMGB1 complexes by endocytosis for incorporation in lysosomal compartments. Intra-lysosomal HMGB1 disrupts lysosomal membranes thereby releasing the HMGB1-transported molecules to stimulate cytosolic sensors that mediate inflammation. This HMGB1-DAMP/PAMP cofactor pathway slowed the development of HMGB1-binding antagonists for diagnostic or therapeutic use. However, recent discoveries that HMGB1 released from neurons mediates inflammation via the TLR4 receptor system, and that cancer cells express fully oxidized HMGB1 as an immunosuppressive mechanism, offer new paths to targeting HMGB1 for inflammation, pain, and cancer.

## 1. Introduction

High mobility group box 1 protein (HMGB1)s a DNA-binding molecule bound to chromatin in all eukaryotic cells [1]. When passively released by dying cells or actively secreted by activated immune and other cells, it is an alarmin and damage-associated molecular pattern molecule (DAMP)**.** In general, alarmins perform distinct intracellular tasks during homeostatic conditions but promote inflammation to initiate repair mechanisms when released extracellularly in response to danger signals [2]. However, exaggerated alarmin responses can increase tissue injury and cause organ dysfunction, a central mechanism in the pathogenesis of acute and chronic inflammatory diseases. Extracellular HMGB1 has been implicated in chemokine, cytokine, metabolic, inflammatory, neuroinflammatory, and anti-inflammatory activities, a diverse range of functions that depend on the molecular binding partners of HMGB1, its extracellular or intracellular location, and its redox state [3,4].

## 2. Extracellular HMGB1 Release

**Figure 1:**
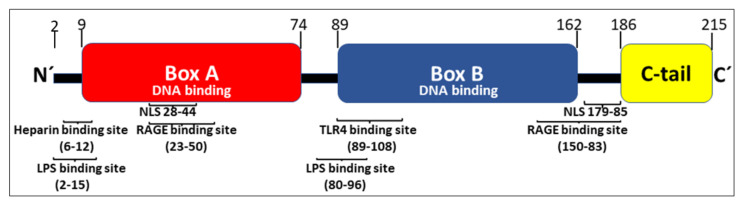
Position of binding sites for HMGB1-receptors, heparin, LPS, and the two nuclear localization sites (NLSs) in the HMGB1 molecule.

HMGB1 expresses 214 amino acids arranged in two consecutive DNA-binding HMG box domains (box A and box B) and an acidic C-terminal tail, containing a stretch of thirty continuous glutamic and aspartic acids (Figure 1).

The two DNA-binding boxes of HMGB1 contain three cysteines and the redox state of each of these residues is critically important for the ability of the nuclear molecule to be secreted and for the extracellular receptor usage. All three cysteines reside in a fully reduced state with thiol groups (all-thiol HMGB1) in inactive cells. Mild HMGB1 oxidation generates disulfide HMGB1 characterized by a disulfide bond between Cys23 and Cys45 while keeping Cys106 in the reduced form. Further oxidation of HMGB1 will produce sulfonyl groups on any or all cysteine residues creating an isoform called sulfonyl HMGB1 [1]. Homo-dimerization of HMGB1 at Cys106 has recently but described to take place both in the nucleus and extracellularly [5]. The biological significance of this molecule needs further investigation.

Active HMGB1 release occurs in several steps. First, nuclear HMGB1 translocates to the cytoplasm, a process that requires JAK-STAT1 signaling that will generate acetylation of critical lysine residues located in the two nuclear localization sites (NLSs) [6,7] (Figure 1). Hyperacetylation of HMGB1 prevents the continuous bidirectional shuttle of HMGB1 between the cytoplasm and the nucleus present in all cells and leads to cytoplasmic accumulation of HMGB1. Nuclear hyperacetylation is also accomplished via increased histone-acetylase (HAT) activity as well as decreased histone-deacetylase (HDAC) activity [7,8,9]. Several agents including metformin, resveratrol, and curcumin (which all enhance sirtuin 1 (SIRT1) deacetylase activity) decrease extracellular HMGB1 release and reduce HMGB1-dependent inflammation [10,11,12,13,14,15,16,17,18]. Decreased activity of SIRT1, a nicotinamide adenine dinucleotide-dependent HDAC, occurs in aging and senescence, suggesting a role for HMGB1 in the inflammation associated with aging (“inflammageing”) [19,20,21]. Additional HDACs including HDAC1 and HDAC4 have likewise been demonstrated to efficiently inhibit active HMGB1 release [8,9,22]. Ethanol reduces HDAC1/4 performance and thus enhances neuronal HMGB1 release [9]. Ischemia-reperfusion injury is another cause of reduced nuclear HDAC1 and HDAC4 activities that generate increased levels of extracellular hyperacetylated HMGB1 [8].

Intranuclear oxidation of HMGB1 to the disulfide isoform is also a prerequisite for the translocation of HMGB1 to the cytosol [23]. Nuclear peroxiredoxins I and II induce an intramolecular disulfide formation between Cys23 and Cys45 to generate disulfide HMGB1 that will be transported out of the nucleus by binding to the nuclear exportin chromosome-region maintenance 1 (CRM1) interacting with the two nuclear export signal sites present in each of the HMG boxes of HMGB1 [7].

**Figure 2:**
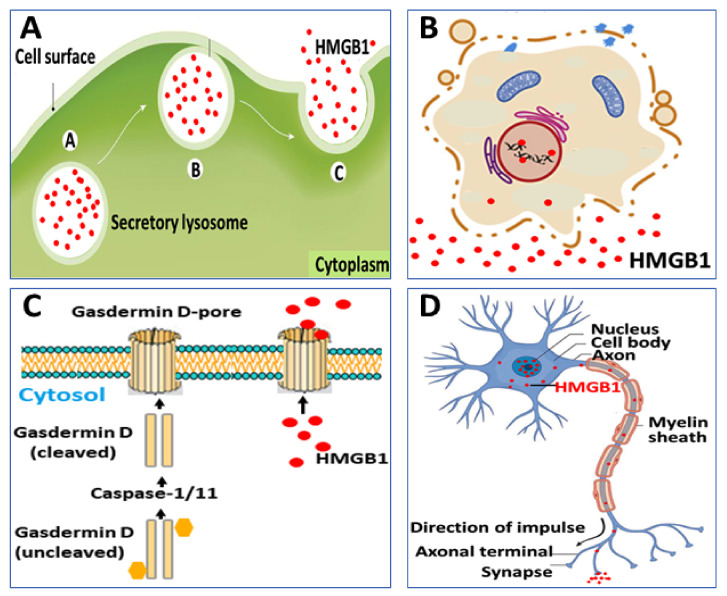
Selected examples of mechanisms for HMGB1 release. (**A**) HMGB1 lacks a secretory signal sequence and is instead packed into secretory lysosomes in hematopoietic cells, before being released extracellularly. (**B**) Pyroptosis and necrosis are both lytic processes that generate HMGB1 release, (**C**) Inflammasome-activated gasdermin D creates pore formation in the outer cell membrane enabling HMGB1 release even before cell lysis may occur. (**D**) Stimulated nociceptors release HMGB1 in a retrograde manner.

Second, cytoplasmic HMGB1 is released extracellularly via several mechanisms. One route proceeds via exocytosis of secretory lysosomes, a pathway also used for IL-1β secretion, although HMGB1 and IL-1β are stored in separate vesicles [24] (Figure 2A). The intracellular events that control the sequestration of cytoplasmic HMGB1 in secretory lysosomes remain to be elucidated. A second route for HMGB1 to exit cells is via its expression on the surface of microparticles derived from activated platelets [25,26]. Vascular injury induces the massive extracellular release of HMGB1 from platelets displaying an important role in the pathogenesis of thrombosis formation and neutrophil activation [27,28,29].

Programmed, proinflammatory cell death (pyroptosis) is an additional mechanism for the regulated release of HMGB1, which is hyperacetylated and expresses the disulfide redox isoform [30,31,32] (Figure 2B,C). This process takes place due to increased caspase-1/caspase-11 activity that generates inflammasome activation and gasdermin D cleavage inducing pore formation and eventually ruptures the outer cell membrane [33]. Gasdermin D-generated nanopore formation and pyroptosis have been implicated as the dominant pathway for HMGB1 release during gram-negative sepsis when caspase-1/caspase-11 double-deficient mice express markedly reduced systemic HMGB1 levels [34]. Recent evidence indicates that stimulated sensory neurons actively secrete HMGB1 in an antidromic fashion by molecular mechanisms that remain elusive [35] (Figure 2D). The neuronally released HMGB1 is most likely disulfide HMGB1 since it acts via TLR4 to mediate inflammation and pain [13,36,37,38,39,40,41,42,43,44,45,46,47] and disulfide HMGB1 is the single redox form of HMGB1 capable of binding to the MD-2/TLR4 receptor complex [48]. Functional consequences regarding the neuronal HMGB1-regulation of inflammation will be discussed later in this review.

Various forms of cell deaths generate passive HMGB1 release expressing different isoforms. Necrosis releases fully reduced, non-acetylated HMGB1 (the habitual nuclear HMGB1 isoform), which acts as a chemotactic factor when bound to CXCL12 generating enhanced CXCR4 signaling [49,50]. Apoptosis causes insignificant extracellular HMGB1 release since the nuclear HMGB1 strongly attached to modified DNA is retained in membrane-sealed apoptotic bodies [51]. However, if the phagocytic clearance of the apoptotic bodies fails this debris may undergo secondary-necrosis and discharge non-acetylated HMGB1 mainly in the sulfonyl redox isoform [52]. As already described, pyroptosis generates disulfide, hyperacetylated HMGB1 [30,31,32].

## 3. HMGB1 Receptor Usage

**Figure 3:**
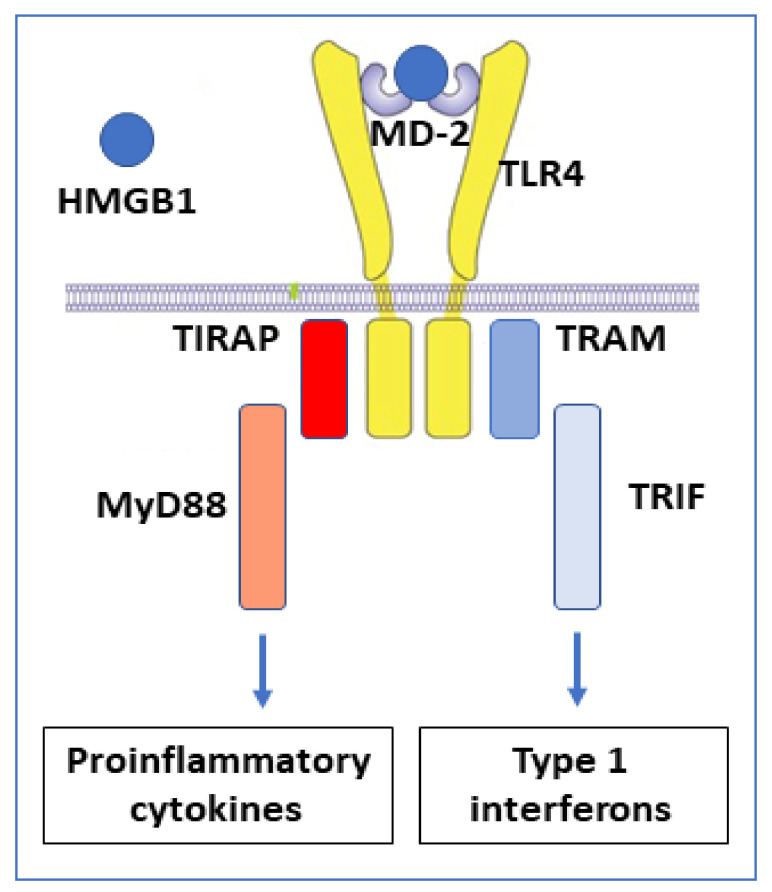
Disulfide HMGB1 binds to MD-2 and activates the TLR4 receptor complex via two separate intracellular signal pathways. Proinflammatory cytokines are formed when the adapter molecule TIRAP gets associated with toll-like receptor 4 and the myeloid differentiation factor 88 (MyD88) that activates the NF-κB signaling pathway. Interferon-β is produced when TRAM (TRIF-related adaptor molecule) associates with TRIF (TIR-domain-containing adapter-inducing interferon-β).

The redox state of HMGB1 determines the ability for receptor interactions and thus the functional outcome of extracellular HMGB1 interactions. TLR4 and the receptor for advanced glycated end-products (RAGE) are the most extensively studied HMGB1 receptors. TLR4 is the HMGB1 receptor causing cytokine and type 1 interferon production [53] (Figure 3). This interaction requires disulfide HMGB1 to bind at low nanomolar avidity to the TLR4 co-receptor MD-2, in an analogous way to LPS, but attaching at another position [48].

**Figure 4:**
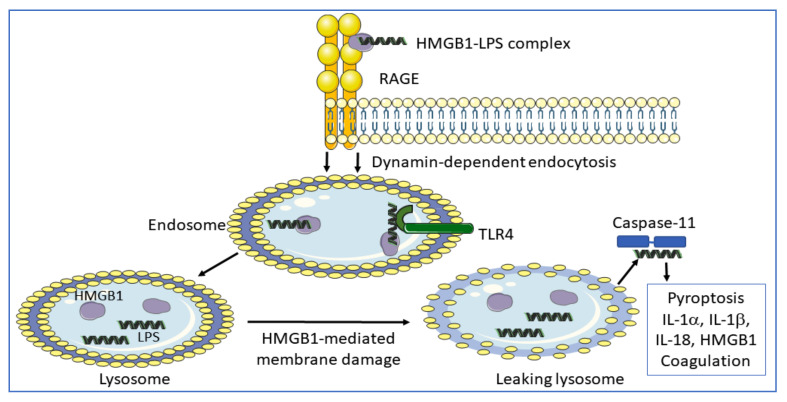
LPS needs HMGB1 to trigger severe inflammation. Injected LPS and type 1 interferon, (which generates HMGB1 release) in TLR4 gene-deficient mice is lethal in contrast to when administered to caspase-11 knockout mice [75,76]. The initial event in LPS toxicity is due to extracellular LPS activation of cell surface TLR4, which triggers extracellular HMGB1 release. HMGB1 has two LPS-binding sites (Figure 1) and thus forms extracellular HMGB1-LPS complexes that get endocytosed via RAGE to finally reach the cytosol culminating in caspase-11 activation (in mice; caspases 4/5 in humans) causing inflammation and coagulation.

RAGE is a multi-ligand receptor expressed by many cell types, predominantly as a preformed intracellular molecule available for rapid NF-κB-controlled translocation to the cell surface [54,55]. RAGE was originally identified as an HMGB1-receptor in the context of studies of neurite outgrowth in the fetal brain without any signs of concomitant proinflammatory activity. This RAGE-binding site in HMGB1 is located in sequence 150–83 [56] (Figure 1). One additional RAGE-binding site situated in the HMGB1 box A domain (sequence 23–50) was later identified and RAGE-interaction with this site has profound proinflammatory effects [57]. Extracellular HMGB1 readily forms heterocomplexes with multiple extracellular DAMPs and PAMPs [58], which are subsequently endocytosed by HMGB1 binding to RAGE for further intracellular transport to the endolysosomal compartment [34,59,60,61,62,63,64,65,66,67,68,69]. Heparin, recombinant truncated HMGB1 box A protein, and acetylcholine each blocks the endocytosis of HMGB1 and its partner molecules [60,70]. HMGB1 heterocomplexes are endocytosed via RAGE expressed on macrophages and finally accumulate in the lysosomal compartment [34] (Figure 4). HMGB1, at high concentration, then accomplishes a unique function inside acidic lysosomes because HMGB1 disrupts the lysosomal membrane at low pH allowing its partner molecules to circumvent degradation and leak into the cytosol. In contrast, any molecule imported into the lysosomal system via antibodies in the absence of HMGB1 is normally degraded there. The HMGB1-imported extracellular DAMPs and PAMPs released from the ruptured lysosomes will subsequently bind and activate cognate cytosolic sensors, a mechanism that would not occur in the absence of the RAGE/HMGB1-assisted transport. Stimulation of proinflammatory cytosolic sensors generates inflammasome activation, pyroptosis, the release of proinflammatory mediators, and activation of the extrinsic coagulation cascade [34,71,72]. Inflammasomes cleave and activate inflammatory caspases such as caspase 1, 4, 5, and 11 resulting in activation of cytoplasmic gasdermin D. The truncated gasdermin D then forms oligomerized molecules producing nanopores in the plasma membrane culminating in pyroptotic cell death (Figure 2C). This process in live and dying cells mediates the release of IL-1α, IL-1β, IL-18, and HMGB1 [30,31,71] (Figure 4). Furthermore, cleaved gasdermin D also activates a membrane-located scramblase inducing phosphatidylserine externalization on the cell surface, where the molecule assembles a complex of cofactor proteases of the coagulation cascade initiating coagulation [33,73,74].

Surprising new findings in tumor biology reveal that oxidized HMGB1 (sulfonyl HMGB1) is an anti-inflammatory molecule that signals via RAGE [77] (Table 1). Sulfonyl HMGB1 has until now been considered as a functionally inert molecule, mainly defined by an absent capacity to generate inflammation, but these new observations implicate sulfonyl HMGB1 in recruiting immunocompetent cells which inhibit cytotoxic cells, thereby impairing their ability to attack and kill the tumor cells. Immunosuppressive cells recruited by sulfonyl HMGB1 include regulatory T lymphocytes (Tregs), M2 macrophages, and myeloid-derived suppressor cells (MDSC). Sulfonyl HMGB1 also downregulates antigen-presenting cells including dendritic cells and plasmacytoid dendritic cells (summarized in Table 1).

**Figure 5:**
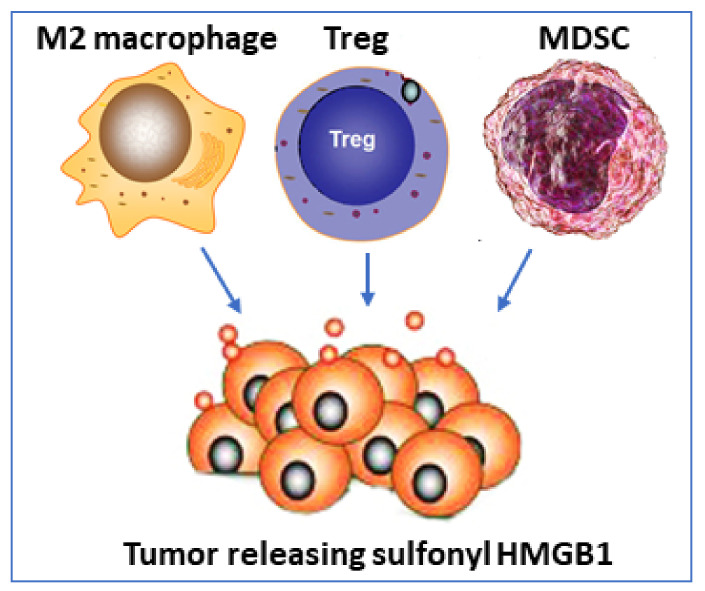
Fully oxidized HMGB1 inhibits cytotoxicity versus tumors. Tumor secreting sulfonyl HMGB1 which attracts M2 macrophages, regulatory T cells, and myeloid-derived suppressor cells (MDSC) which all inhibit a cytotoxic cell response against the tumor.

Administration of HMGB1 inhibitors improved outcomes from cancer in several experimental models. Anti-HMGB1 therapy inhibited tumor growth, diminished the recruitment of immunosuppressive cells, and enhanced the antigen-presenting capacity of tumor-associated dendritic cells [77]. Furthermore, the therapeutic efficacy of checkpoint immune inhibitors was enhanced when concomitant HMGB1 blocking treatment was provided. These results suggest a very intriguing scenario where sulfonyl HMGB1 may occupy a supportive role in the resolution of inflammation, but a detrimental role in the defense against tumors. The results suggest further studies of the mechanism are warranted to determine the role of sulfonyl HMGB1/ RAGE-dependent biology in cancer because it offers an interesting experimental therapeutic strategy (Figure 5).

**Figure 6:**
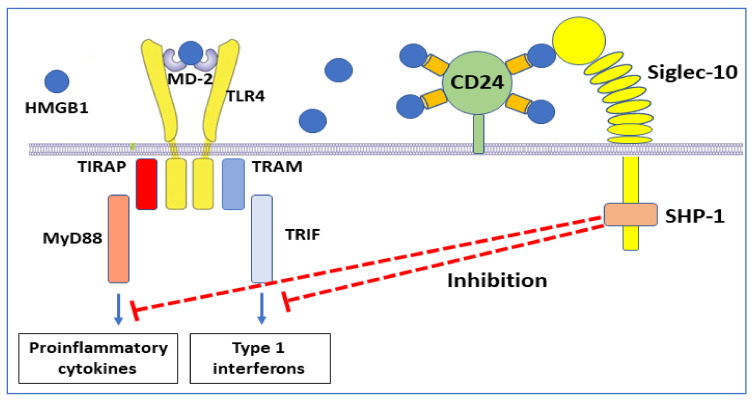
An anti-inflammatory HMGB1-dependent pathway. A trimolecular complex formed by HMGB1, CD24, and Siglec-10 generates intracellular cell signaling that turns off NF-κΒ-dependent inflammatory processes.

The immunosuppressive regulation of sulfonyl HMGB1 via RAGE is not the only HMGB1 anti-inflammatory mechanism [77]. It has previously been demonstrated that HMGB1, of undefined redox isoform, binds to CD24 (Table 1) [78]. This cell surface sialoglycoprotein is expressed by several cell types including dendritic cells, where it provides costimulatory signals to T cells but lacks a mechanism for signal transduction. HMGB1-CD24 forms a trimolecular complex on dendritic cells with the signaling receptor Siglec-10, which subsequently associates with the tyrosine phosphatase SHP-1, a negative regulator of nuclear factor-kB (NF-κΒ) activation [78] (Figure 6).

The consequence of these molecular events is thus downregulated inflammation. Experimental administration of CD24-Fc fusion protein inhibited inflammation in preclinical models of virus infection, autoimmunity, and graft-versus-host disease [79,80,81]. Clinical studies based on the administration of soluble CD24 and CD24Fc are in progress for patients with severe COVID-19 [82].

## 4. Sensory Neurons Direct Inflammation via HMGB1 Release

**Figure 7:**
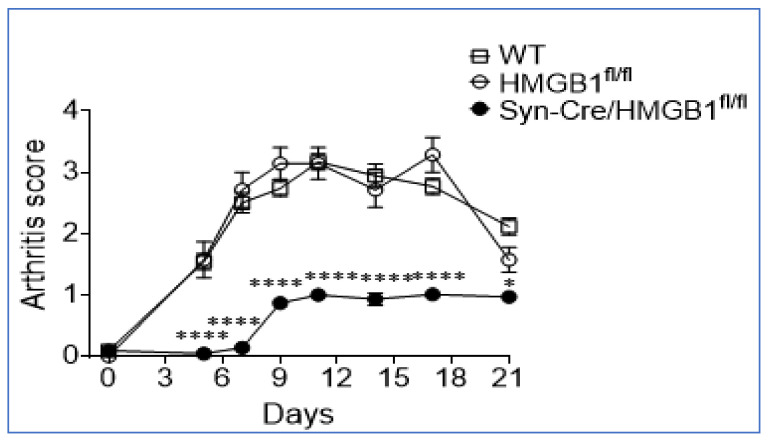
Ablation of neuronal HMGB1 reduces joint inflammation. Polyarthritis was induced by the administration of anti-collagen antibodies in mice. Wild type (WT) and HMGB1^fl/fl^ control mice developed severe polyarthritis. Significantly delayed onset and reduced severity of polyarthritis were observed in neuronally HMGB1 gene-deficient mice (Syn-Cre/HMGB1^fl/fl^). *: *p <* 0.05, ****: *p <* 0.0001 vs. HMGB1^fl/fl^ control. Reproduced from Yang et al. [35].

Sensory neurons, termed “nociceptors” mediate neuroinflammation through the retrograde or “antidromic” release of neuropeptides, neurotransmitters, and incompletely defined mediators. Recently we reported that HMGB1 is a necessary and sufficient mediator of neuroinflammation because nociceptors harvested from transgenic mice expressing channelrhodopsin-2 (ChR2) directly release HMGB1 when stimulated by light [35]. In collagen antibody-induced arthritis in mice, ablation of neuronal HMGB1 decreased hyperalgesia, delayed onset, and reduced intensity of joint inflammation and cartilage destruction compared to wild type (WT) or HMGB1 floxed (*HMGB1^fl/fl^*) control mice (Figure 7).

**Figure 8:**
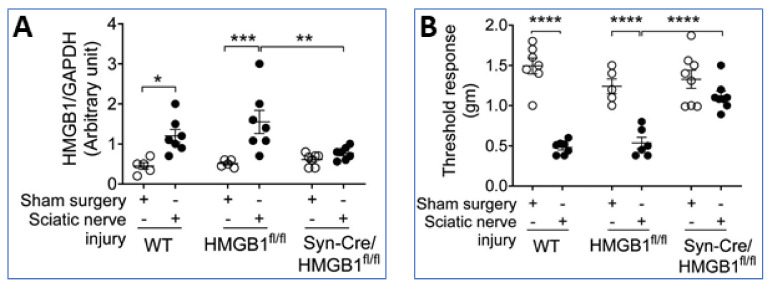
Ablation of neuronal HMGB1 reduces inflammation and hyperalgesia after sciatic nerve injury. Standardized sciatic nerve injury was induced via nerve ligation. (**A**) HMGB1 levels were significantly increased in paw tissue from WT and HMGB1^fl/fl^ control mice in contrast to Syn-Cre/ HMGB1^fl/fl^ mice (* *p <* 0.05, ** *p <* 0.01, *** *p <* 0.001). (**B**) Mechanical sensitivity assessed using von Frey filaments and the Dixon up-down method to calculate the threshold response demonstrated that hyperalgesia after sciatic nerve injury was significantly reduced in Syn-Cre/HMGB1^fl/fl^ mice as compared to HMGB ^fl/fl^ control mice (**** *p <* 0.0001). Reproduced from Yang et al. [35].

Furthermore, sterile sciatic nerve injury produces inflammation, swelling, and hyperalgesia in the paws of wild type mice (WT) and HMGB1 floxed *HMGB1^fl/fl^* mice, but these responses are attenuated in neuronal-specific HMGB1 knock-out (Syn-Cre/HMGB1^fl/fl^) mice (Figure 8A,B) [35]. These and other results indicate neuronal HMGB1 is required to mediate nerve injury-induced tissue inflammation and neuropathic pain.

The redox state of the neuronally released HMGB1 is yet to be defined, but it is likely the disulfide form because neuroinflammation and hyperalgesia are TLR4-dependent, and disulfide HMGB1 is a specific TLR4 ligand [83,84,85,86]. Neuronal TLR4 KO mice are also significantly protected from sciatic injury-induced allodynia and skin inflammation [87]. Other studies of global TLR4 knockout mice likewise indicate that TLR4 is required for HMGB1-mediated hyperalgesia [83,84].

Active neuronal HMGB1 release is not restricted to peripheral sensory nerves but has been demonstrated to occur in yet undefined neurons in the central nervous system too [9,46,88,89,90]. Cultured primary cortical neurons stimulated by TNF release HMGB1 [88]. Ethanol triggers HMGB1 release from neurons in rat hippocampal-entorhinal cortex brain slice cultures [9], as ethanol reduces HDAC activity which promotes the release of acetylated HMGB1. Targeting neuronal HMGB1 reduces the expression of TNF and IL-1β in microglia cells in the cultured brain slices. Hyperexcitatory brain neurons from Alzheimer’s patients also release HMGB1, which binds to TLR4 and mediates neurite degeneration [46]. A recently developed HMGB1-specific mAb blocking the TLR4-binding epitope of HMGB1 has demonstrated beneficial therapeutic effects in mouse models of preclinical Alzheimer´s disease [46,91], and other neutralizing anti-HMGB1 mAbs exerted neuroprotection in a rat model of Parkinson´s disease [89]. In the anti-HMGB1 mAb-treated group, HMGB1 was retained in the nucleus of neurons and astrocytes, whereas in the control mAb-treated group cytoplasmic HMGB1 translocation was observed in both neurons and astrocytes.

In summary, these multiple observations suggest that HMGB1 is actively released during neuronal depolarization and plays a key etiologic role in the initiation and amplification of inflammation.

## 5. HMGB1 in COVID-19

There are presently almost 200,000 publications about COVID-19 listed on PubMed but only 40 of them investigated the role of HMGB1, out of which only 4 reports on elevated systemic HMGB1 levels in COVID-19 patients [92,93,94,95]. This is a remarkably small number considering that extensive necrosis and hyperinflammation in the disease should generate substantial HMGB1 release. A hyperexcited HMGB1-RAGE axis would also be expected since the respiratory tract macrophages, epithelial, and endothelial cells release large amounts of extracellular HMGB1, and its cognate receptor RAGE is constitutively abundantly expressed in the lungs only. It is therefore highly surprising that only a few papers are documenting robustly increased systemic amounts of HMGB1 during the acute stage of severe COVID-19. The HMGB1 ELISAs used in the four reports that demonstrated high HMGB1 levels included antibodies with different specificities for HMGB1 than those applied in standardized HMGB1 ELISAs used in the majority of HMGB1 studies. It is most likely that these four papers reflect COVID-19 pathophysiology. We further speculate that during the acute stage of the disease large amounts of extracellular endogenous DNA and other DAMPs are released by extensive cell death. This combined with extracellular viral RNA and other PAMPs bound to HMGB1 may interfere with HMGB1 assays. Standard HMGB1 ELISA methods commonly include buffer steps to dissociate HMGB1 and partner molecules bound to HMGB1 enabling the ELISA antibodies to recognize HMGB1. Based on our unpublished results we suspect that some standardized HMGB1 ELISAs do not perform accurately with COVID-19 plasma samples and fail to remove complex-bound molecules efficiently from HMGB1, which produces confounding results.

**Figure 9:**
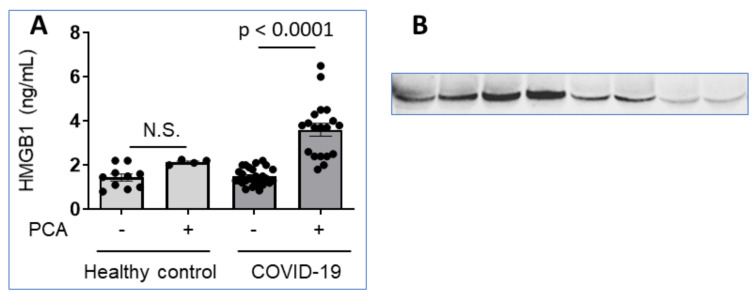
Plasma HMGB1 levels are increased in patients with severe COVID-19. (**A**) Multiple plasma samples from COVID-19 patients (27 samples from 9 patients) and healthy controls (14 samples from 4 healthy controls) were analyzed in HMGB1 ELISA (IBL International GmbH, Germany). The samples were either pretreated by perchloric acid (PCA) or not [96]. (**B**) Plasma samples from two COVID-19 patients and two healthy controls were subjected to SDS polyacrylamide gel electrophoresis in reducing conditions and probed with a monoclonal anti-HMGB1 antibody. Lanes 1-4 shows plasma samples from day 0, 2, 3, 4 from the admission of a patient with lethal Covid-19 infection; lanes 5–6 represent samples from a severely ill COVID-19 patient on day 0 and 1 from admission, while lanes 7–8 demonstrate results in two healthy controls.

This view is supported by our recent analysis of plasma samples from 9 COVID-19 patients with severe hyperinflammation using ELISA methods that revealed HMGB1 levels within the normal range (Figure 9A). However, immunoblotting analysis under reducing conditions of the same samples demonstrated highly elevated HMGB1 levels as compared to normal controls indicating pathologically increased plasma concentrations (Figure 9B). Pretreatment of the plasma samples with perchloric acid [96] to dissociate molecules attached to HMGB1 shifted the ELISA results to demonstrate increased, pathological HMGB1 levels, despite that the harsh acidic handling partly damaged the samples (Figure 9A). Taken together, it seems that there are exceptional problems regarding systemic HMGB1 quantification during acute COVID-19, due to yet undefined partner molecules attaching strongly to HMGB1 and causing steric hindrance for antibody recognition.

ELISA measurement of systemic HMGB1 levels in some other clinical conditions including active systemic lupus erythematosus (SLE) or septic shock has previously been reported to underestimate results reminding of the problems that we encountered in our pilot study in COVID-19 patients. Barnay-Verdier et al. studied HMGB1 quantification using ELISA in plasma samples from patients with septic shock [96]. They compared results in plasma versus plasma subjected to perchloric acid exposure prior to ELISA. The results were straightforward, PCA-ELISA detected significantly higher amounts of HMGB1 in plasma samples compared to conventional ELISA. Another study unexpectedly found that lupus patients with active disease had HMGB1 levels measured by ELISA to be at the same or even at lower levels than in healthy controls [97]. In contrast, western blot assessment demonstrated huge differences between healthy controls and patients with active lupus, who expressed high HMGB1 levels. The plasma molecules that presumably bound and masked HMGB1 in ELISA measurements were not identified.

## 6. HMGB1 and Acetylcholine-Potent Antagonists Balancing Inflammation

Over the past 20 years, amazingly consistent and successful results from preclinical and in vitro studies have revealed that acetylcholine is a strong inhibitor of HMGB1-provoked inflammation and pain [60,98,99,100,101,102,103,104,105,106,107,108,109,110]. The two ancient molecules acetylcholine and HMGB1 have during evolution formed a functional yin-yang relationship. Homeostasis in inflammation is obtained when the functional influences mediated by HMGB1 and acetylcholine are in balance. Therapeutic results in preclinical studies using vagus nerve stimulation, choline esterase inhibitors, or α7-nicotinic acetylcholine receptor (α7nAChR) agonists are strikingly similar to those seen after HMGB1-specific blocking treatment regarding kinetics and final outcome [3,111,112,113,114]. Acetylcholine inhibits HMGB1 release [102,103,105,106,107,115,116,117], TLR4/MyD88/NF-κB signaling [118], and RAGE-mediated endocytosis of HMGB1 and HMGB1 complex-bound to DAMPs or PAMPs [60]. Each one of these inhibitory accomplishments is beneficial for controlling HMGB1-mediated inflammation. SIRT1 functions were enhanced by α7nAChR-specific agonist stimulation and inhibited by an α7nAChR- specific antagonists supporting a role for α7nAChR signaling to mediate increased SIRT1 activity inhibiting HMGB1 release [119]. Furthermore, electroacupuncture pretreatment using a specific acupoint termed ST36 attenuated acute lung injury through α7nAChR-mediated inhibition of HMGB1 release in rats after cardiopulmonary bypass [107]. It was recently demonstrated that low-intensity electroacupuncture stimulation of the ST36 acupoint excited PROKR2-expressing sensory neurons to activate the cholinergic anti-inflammatory system [116].

Enhancing nuclear HMGB1 deacetylation to inhibit the nucleocytoplasmic translocation and subsequent extracellular release may thus offer a promising treatment for HMGB1-mediated inflammation. This insight should encourage further clinical studies using non-invasive transcutaneous auricular vagus nerve stimulation or electroacupuncture stimulation at carefully selected acupoints to treat uncontrolled HMGB1-triggered inflammation and neuropathic pain [101,120]. These α7nAChR-mediated therapeutic means augment SIRT1 function and thus inhibit HMGB1 release and subsequent inflammation. It is thus conceivable that HMGB1 antagonists and cholinergic anti-inflammatory activation generate almost interchangeable results in preclinical treatment studies since acetylcholine inhibits extracellular HMGB1 release.

## 7. Key Challenges in the HMGB1 Field

Even though extracellular HMGB1 has been intensely studied for more than two decades there are basic methodological shortcomings that need urgent attention. We need improved methods to quantify extracellular levels of HMGB1 isoforms in clinical samples. The problem of steric hindrance generated by molecules complex-bound to HMGB1 in vivo has here been exemplified in the context of COVID-19, SLE, and septic shock and must be resolved via the invention of improved tools for diagnostic and therapeutic purposes. There are presently no existing methods to quantify HMGB1 redox isoforms or other posttranslational modifications. These obstacles severely delay a further exploration of the fascinating and important biology created by extracellular HMGB1.

Despite numerous successful preclinical therapeutic studies with HMGB1 antagonists in inflammation and pain conditions there is still no clinically approved treatment targeting HMGB1 specifically, which is both disappointing and inspiring. The HMGB1 protein expresses 99% identity among mammals, which should facilitate the process, while on the other hand molecules attached to the extracellular HMGB1 complicate the development of HMGB1-binding antagonists, especially when HMGB1-RAGE endocytosis needs to be targeted. One academic research group in Japan has generated an anti-HMGB1 mAb recognizing the repetitive C-terminal part of the molecule, which conceivably might be an element less engaged by partner molecules in vivo [89,121,122,123,124,125,126,127,128,129,130,131,132]. This antibody has exhibited impressive therapeutic efficacy in many animal models of neuroinflammation both in the central nervous system and in the periphery, most of which events are TLR4-dependent. Another Japanese research group recently reported a successful creation of an HMGB1-specific mAb blocking the HMGB1 sequence engaged in MD-2/TLR4 interaction. This antibody impeded HMGB1-mediated TLR4-dependent biological effects in vitro and exerted beneficial therapeutic effects in a preclinical model of Alzheimer´s disease [91]. These are indeed encouraging examples to guide and inspire further clinical development of HMGB1-targeted therapy.

## Figures and Tables

**Table 1:** Extracellular HMGB1 redox forms determine functional outcomes in inflammation.

HMGB1 Redox Form	Partner Molecule	Receptor	Biological Response	Reference
All-thiol	CXCL12	CXCR4	Chemotaxis	[50]
Disulfide	None	TLR4	Cytokines	[53]
Sulfonyl	Unknown	RAGE	Accumulation of Tregs and MDSCs, enhanced M2/M1 macrophage ratio and dendritic cell tolerogenicity	[77]
Undetermined	Many PAMPs and DAMPs	RAGE	Inflammasome activation, hyperinflammation, coagulation, pyroptosis	[34]
Undetermined	None	CD24+ Siglec-10	NF-κΒ inhibition	[78]

## Data Availability

Raw data available upon request to corresponding author.

## Abbreviations

HMGB1	high mobility group box 1 protein
RAGE	receptor for advanced glycation end-product
TLR4	toll-like receptor 4
MD-2	myeloid differentiation factor 2
TIRAP	TIR domain containing adaptor protein
TRAM	toll-receptor-associated molecule
MyD88	myeloid differentiation primary response 88
TRIF	TIR-domain-containing adapter-inducing interferon-β
CXCL12	C-X-C Motif Chemokine Ligand 12
CXCR4	C-X-C chemokine receptor type 4
NF-κB	nuclear factor kappa-light-chain-enhancer of activated B cells
LPS	lipopolysaccharide
DAMP	damage-associated molecular pattern molecule
PAMP	pathogen-associated molecular pattern molecule
HDAC	histone-deacetylase
HAT	histone-acetylase
SIRT1	sirtuin 1
CRMI	nuclear exportin chromosome-region maintenance 1
NLS	nuclear localization site
SHP-1	Src homology 2 domain-containing protein tyrosine phosphatase 1
a7nAChR	a7-nicotinic acetylcholine receptor
MDSC	myeloid-derived suppressor cells
Treg cell	regulatory T lymphocyte
M1 macrophage	proinflammatory macrophage
M2 macrophage	anti-inflammatory macrophage
Syn-Cre/HMGB1^fl/fl^ mice	neuronally HMGB1 gene-deficient mice
CD24	cluster of differentiation 24
Siglec-10	Sialic acid-binding Ig-like lectin 10
